# Post-Exercise Increase in Maximal Voluntary Ventilation in Swimmers and Water Polo Players: Different Changes in Spirometric Indices and Inspiratory Flow–Volume Loop Parameters

**DOI:** 10.3390/jcm15218525

**Published:** 2026-11-02

**Authors:** Małgorzata Stachowicz, Mateusz Żbikowski, Aleksandra Stachowicz, Michał Stachowicz, Grzegorz Wójcik

**Affiliations:** 1 Department of Water and Winter Sports, Józef Piłsudski University of Physical Education in Warsaw, ul. Marymoncka 34, 00-968 Warsaw, Poland; 2 Department of Intelligent Systems and Data Science, Polish-Japanese Academy of Information Technology, 02-008 Warsaw, Poland; 3 Doctoral School, Józef Piłsudski University of Physical Education in Warsaw, ul. Marymoncka 34, 00-968 Warsaw, Poland; 4 Collegium Medicum, Nicolaus Copernicus University in Toruń, ul. Jagiellońska 13–15, 85-067 Bydgoszcz, Poland; 5 Department of Neuroinformatics and Biomedical Engineering, Institute of Computer Science, Maria Curie-Skłodowska University, 20-033 Lublin, Poland

**Keywords:** maximal voluntary ventilation, spirometry, flow–volume loop, post-exercise bronchodilation, swimming, water polo

## Abstract

**Background/Objectives:** Maximal voluntary ventilation (MVV) is often calculated from the forced expiratory volume in one second (FEV_1_ × 35–40), assuming a constant relationship between the two. It is unknown whether this holds immediately after maximal exercise, or whether post-exercise changes in MVV are similar across disciplines with different breathing patterns. The aim was to compare post-exercise changes in MVV and flow–volume loop indices in swimmers and water polo players, and to explore whether the change in FEV_1_ predicts the change in MVV in individual athletes. **Methods:** The study included 29 competitive swimmers and 33 competitive water polo players (spirometry performed in 61 persons), examined in the competitive period of the annual training cycle. Spirometry (14 variables, including inspiratory loop indices and forced expiratory time) was performed at rest and immediately after 30 s of maximal tethered swimming (the post-exercise measurement was completed within approximately 2 min). Nonparametric longitudinal analysis of variance (F1-LD-F1 design, ANOVA-type statistic) was used. For each person the error of the relative change in MVV estimated from FEV_1_ (change in MVV − change in FEV_1_) was calculated. **Results:** MVV increased in both groups (median change: swimmers 7 percentage points, water polo players 17 percentage points; time effect *p* < 0.001). The increase was larger in water polo players (group × time interaction *p* = 0.048), whose resting MVV was lower, but after adjustment for resting MVV the difference in the increase was not significant (*p* = 0.319). The change in FEV_1_ did not predict the change in MVV in swimmers (ρ = 0.00; *p* = 0.98), and in water polo players the association was moderate (ρ = 0.44; *p* = 0.012). The FEV_1_-based estimate of the change in MVV overestimated the increase in swimmers (median error −5.9 percentage points; 95% CI −8.0 to −4.5) and underestimated it in water polo players (11.9 percentage points; 95% CI 7.7 to 13.7); the between-group difference was *p* < 0.001, but it was not significant after adjustment for age and body mass index (BMI; *p* = 0.112). In swimmers, FEV_1_ and inspiratory loop indices (forced inspiratory vital capacity, FIVC; forced inspiratory volume in one second, FIV1; peak inspiratory flow, PIF) increased, but their resting values indicated incomplete inspiratory effort in the baseline measurement; in water polo players forced expiratory time shortened to a greater extent. **Conclusions:** A post-exercise increase in MVV occurs in both disciplines, but it is accompanied by different changes in spirometric indices. In the early post-exercise period (within approximately 2 min), the change in FEV_1_ does not reflect the change in MVV in individual athletes, and the direction of the error of the estimate differs between the groups, which limits the usefulness of MVV calculated from FEV_1_ in this period. The results are exploratory and require confirmation in a larger sample balanced for sex and age.

## 1. Introduction

Maximal voluntary ventilation (MVV) is the largest volume of air that a person can ventilate in one minute during breathing that is as fast and as deep as possible. In pulmonary function diagnostics and in exercise testing, MVV is often not measured but calculated as the product of forced expiratory volume in one second (FEV_1_) and a factor of 35–40 [1]. This procedure assumes that ventilatory capacity depends mainly on bronchial patency. However, agreement between measured and calculated MVV is only moderate already at rest, both in healthy people and in patients with chronic obstructive pulmonary disease [2]. Separate prediction equations based on FEV_1_ have also been developed for athletes [3].

Immediately after exercise the relationship between FEV_1_ and MVV may change. In healthy people exercise induces bronchodilation, the frequency and magnitude of which depend on age and baseline airway patency and do not differ between women and men [4,5,6]. Exercise also changes the function of the respiratory muscles. Inspiratory muscle fatigue has been found after swimming exercise [7,8], and the work of breathing during maximal exercise affects the distribution of blood flow between the respiratory and locomotor muscles [9,10]. It cannot therefore be assumed that the post-exercise change in MVV will be proportional to the change in FEV_1_.

Competitive swimmers have larger lung volumes than athletes of other disciplines and recreationally active people, and during maximal exercise, they increase ventilation mainly by increasing tidal volume [11,12,13]. Indices of respiratory function also differ between athletes of other disciplines, including team games [14,15]. Swimming and water polo are practiced in the same environment, but they require a different way of breathing. In swimming, inspiration is short, performed in a specific phase of the movement cycle and repeated with high frequency. In water polo, breathing is irregular and interrupted by periods of breath holding during play. Comparison of the two groups after an identical exercise stimulus makes it possible to assess whether post-exercise changes in MVV and spirometric indices are similar in both groups or differ between them.

The aim of the study was to compare post-exercise changes in MVV and in the indices of the expiratory and inspiratory flow–volume curve in competitive swimmers and water polo players. It was hypothesized that MVV would increase after exercise in both groups, but that the accompanying changes in spirometric indices would differ between the disciplines. In addition, in an exploratory analysis, it was assessed whether the change in FEV_1_ makes it possible to predict the change in MVV in individual athletes; that is, whether MVV calculated from FEV_1_ would reflect the post-exercise increase in measured MVV.

## 2. Materials and Methods

### 2.1. Participants

The study included 29 competitive swimmers (18 men and 11 women) and 33 competitive water polo players (men only). The characteristics of the participants are presented in Table 1. Both groups were examined in the same phase of the annual training cycle, i.e., in the competitive period, in which training is aimed at achieving peak competitive performance; thus, the swimmers and water polo players were assessed at a comparable stage of training preparation. The swimmers were examined in several measurement sessions during the annual training cycle; the present analysis includes only the session conducted in the competitive period, in which the water polo players were also examined. From one water polo player only anthropometric data were obtained, so 32 water polo players were included in the analysis (61 persons in total), and the number of complete pairs of measurements ranged from 15 to 32 depending on the variable. Persons without chronic respiratory diseases (including diagnosed obstructive airway diseases such as asthma), without a diagnosed allergic disease, and without symptoms of respiratory tract infection on the day of the examination were included in the study. This was established on the basis of an interview conducted before the spirometric examination, during which participants were asked about diagnosed asthma or other obstructive airway diseases, allergic diseases, the use of anti-asthmatic medication, and current symptoms of respiratory tract infection; none of the participants reported a diagnosis of asthma or other obstructive airway disease or the use of anti-asthmatic medication. The study was approved by the Bioethics Committee of the Józef Piłsudski University of Physical Education in Warsaw (KB/AWF/003/2025). All participants gave written consent to take part in the study, which was conducted in accordance with the Declaration of Helsinki.

### 2.2. Exercise Protocol

The athletes performed 30 s of tethered front crawl swimming at maximal intensity in a 25 m indoor swimming pool (water temperature 27–28 °C), according to the protocol described previously [16]. The thrust force was recorded continuously with a MAX-6 dynamometric system with a WTP-003 amplifier (Staniak, Warsaw, Poland), and the maximal thrust force (Fmax, N) was determined. The protocol, equipment and test conditions were the same in both groups, which made it possible to compare the spirometric response to an identical exercise stimulus.

### 2.3. Spirometric Examination

Spirometry was performed with a Spirolab III spirometer (MIR, Rome, Italy) in accordance with the American Thoracic Society/European Respiratory Society (ATS/ERS) recommendations [17]—at rest and immediately after the end of exercise. Forced vital capacity (FVC), forced expiratory volume in one second (FEV_1_), the FEV_1_/FVC ratio, peak expiratory flow (PEF), maximal expiratory flows after exhalation of 25%, 50%, and 75% of FVC (FEF25, FEF50, FEF75), the FEV_1_/FEV6 and FEV3/FVC ratios, and forced expiratory time (FET) were assessed. Forced inspiratory vital capacity (FIVC), forced inspiratory volume in one second (FIV1), and peak inspiratory flow (PIF) were read from the inspiratory flow–volume loop. MVV was measured directly in a 12 s maximal voluntary ventilation test. All values were expressed as percentages of predicted values, which the spirometer software determined individually, taking into account sex, age, body height, and ethnic origin; for the expiratory indices the Global Lung Function Initiative equations were used [18]. Normalization to predicted values made possible a joint analysis of women and men and a comparison of groups differing in sex composition and age. For this reason, all indices, including the FEV_1_/FVC ratio, are presented in % of predicted value. Because persons with diagnosed obstructive airway diseases were not included in the study (Section 2.1), FEV_1_/FVC was analyzed as an index of the post-exercise response and not for the diagnosis of airway obstruction. One resting FEF25 value in a swimmer (3% and 12% of the predicted value in consecutive trials) was considered a measurement artifact and was excluded from the analysis of this variable.

The same measurement procedure was used at rest and after exercise. The spirometer was placed at the edge of the swimming pool. Immediately after the end of the 30 s tethered swimming test, the participant left the water, quickly dried face and hands with a towel, and started the spirometric test approximately 20–30 s after the end of exercise. In accordance with the manufacturer’s instructions for the FVC test (Spirolab III User Manual, MIR, Rome, Italy), in a single recording and without removing the mouthpiece, the participant performed three tidal breaths, a maximal inhalation, a forced exhalation, and a rapid maximal forced inhalation (inspiratory flow–volume loop); after three further tidal breaths the cycle was repeated, so that at least three (three to four) forced maneuvers were obtained in one test. The spirometer software selected the best maneuver (the highest sum of FVC and FEV_1_) in accordance with the ATS/ERS acceptability and repeatability criteria [17]. The MVV test was performed immediately after the FVC test, and the whole post-exercise measurement was completed within approximately 2 min after the end of exercise. This time window covers the early phase of the respiratory response, in which post-exercise bronchodilation is observed [4,6], and precedes the period in which exercise-induced bronchoconstriction usually develops (5–15 min after exercise) [19]. Vital capacity (VC) was measured only at rest, because a slow VC maneuver cannot be performed correctly immediately after maximal exercise. Before the measurements, each participant received a detailed explanation of how to perform the maneuvers and had the opportunity to practice them; all participants had undergone spirometry before. The MVV test (12 s) was performed once at rest and once after exercise, because repeated maximal voluntary ventilation maneuvers could induce symptoms of hyperventilation (hypocapnia, dizziness) and respiratory muscle fatigue, particularly immediately after maximal exercise.

### 2.4. Statistical Analysis

Because of deviations of the distributions from normality and the presence of outliers, the data are presented as median and interquartile range (Q1–Q3). The effect of exercise was assessed using nonparametric analysis of variance for longitudinal data in the F1-LD-F1 design [20,21]. The between-subject factor was group (swimmers, water polo players) and the within-subject factor was the time point (rest, post-exercise). For the effects of group, time, and the group × time interaction, the ANOVA-type statistic (ATS) was calculated. Relative treatment effects (RTE) were determined as the measure of effect; an RTE value greater than 0.5 means results higher, and a value smaller than 0.5 means results lower than the average for the whole sample. Specific comparisons were performed with the Wilcoxon signed-rank test (rest and post-exercise measurement in each group) and the Mann–Whitney *U* test (swimmers and water polo players at each time point), applying the Holm correction for four comparisons within a variable [22]. The post-exercise increase (Δ) was calculated as the difference between the post-exercise value and the resting value (in percentage points, pp). For FEV_1_ and MVV, the relative change after exercise was additionally calculated—(post-exercise value − resting value)/resting value × 100%. The MVV/FEV_1_ ratio at rest and after exercise was also calculated. Because both values were expressed as percentages of predicted values, this ratio reflects the change in the relationship between MVV and FEV_1_ in the same person and not its absolute level. The change in the ratio was assessed with the Wilcoxon test in each group, and the difference in changes between the groups with the Mann–Whitney *U* test. The effect of resting MVV on its increase was assessed with a linear regression model in which the dependent variable was the change in MVV, and the explanatory variables were group and resting MVV (HC3 robust standard errors). Persons with both measurements complete were included in the analysis of each variable. The significance level was set at α = 0.05. All analyses were performed in the Python 3.12 environment (https://www.python.org; SciPy, https://scipy.org; statsmodels, https://www.statsmodels.org; matplotlib, https://matplotlib.org); the ATS procedure was implemented in accordance with the nparLD package [21] and verified against the results of that package.

The agreement of the changes in MVV and FEV_1_ was assessed in an exploratory analysis, not planned before data collection. Because MVV, calculated as the product of FEV_1_ and a constant factor, changes proportionally to FEV_1_, for each person the relative change in MVV and the relative change in FEV_1_—(post-exercise value − resting value)/resting value × 100%—and their difference were calculated; the difference was called the error of the relative change in MVV estimated from FEV_1_ (a negative value means that FEV_1_ overestimates the increase in MVV, a positive value that it underestimates it). The association of the changes in MVV and FEV_1_ was assessed with the Spearman coefficient, the error was described by the median [Q1–Q3] with the 95% confidence interval (CI) of the median (bootstrap, 10,000 samples) and by the limits of agreement (mean ± 1.96 SD), and the difference in the error between the groups with the Mann–Whitney *U* test. The dependence of the increase in MVV on the change in FEV_1_ was assessed by linear regression with group and resting MVV (HC3 robust standard errors). Because the groups differ in sex and age, a sensitivity analysis limited to men, a comparison of the sexes among swimmers, and a model of the error with group, age, and body mass index (BMI) were performed. Apart from the specific comparisons (Holm correction within a variable), these analyses have no correction for multiple testing and are exploratory in character.

## 3. Results

### 3.1. Indices of the Expiratory Flow–Volume Curve and MVV

FVC, PEF, and FEF25 did not change significantly after exercise (time effect: *p* = 0.752, *p* = 0.633, and *p* = 0.749), and the group × time interaction was not significant for these variables (Table 2). FEV_1_ increased after exercise (time effect *p* < 0.001), and the interaction was significant (*p* = 0.033). The increase in FEV_1_ was significant in swimmers (median change 10 pp; *p* < 0.001) but not in water polo players (5.5 pp; *p* = 0.121). FEV_1_/FVC, FEF50, and FEF75 changed significantly over time (*p* = 0.042, *p* = 0.002 and *p* < 0.001) without a significant interaction. In the specific comparisons the increase in these indices was significant in swimmers, and in water polo players it concerned only FEF75 (*p* = 0.030). The values of FEV_1_/FVC, FEF25, FEF50, and FEF75 were higher in swimmers (group effect: *p* from < 0.001 to 0.027).

MVV increased significantly in both groups: in swimmers from 123% to 132% of the predicted value (*p* = 0.001) and in water polo players from 106% to 120% (*p* = 0.001). Among the variables presented in Table 2, the time effect was the largest for MVV (ATS = 30.87; *p* < 0.001). Swimmers had a higher MVV than water polo players at rest (*p* < 0.001) and after exercise (*p* = 0.046), but the increase was larger in water polo players (median change 17 and 7 pp; interaction *p* = 0.048), as a result of which the difference between the groups decreased. After adjustment for resting MVV the difference in the increase between the groups was not significant (4.7 pp; 95% CI from −4.6 to 14.0; *p* = 0.319), which indicates that the larger increase in water polo players was associated mainly with the lower baseline value. The median relative change in MVV was 5.6% in swimmers and 17.2% in water polo players, and that in FEV_1_ was 10.2% and 4.8%, respectively.

The MVV/FEV_1_ ratio decreased after exercise in swimmers from 1.16 (1.05–1.21) to 1.10 (1.00–1.18) (*p* = 0.018), and in water polo players it increased from 0.95 (0.86–1.01) to 1.03 (0.95–1.15) (*p* = 0.004). The change in this ratio differed between the groups (*p* < 0.001).

### 3.2. Indices of the Inspiratory Flow–Volume Loop and Expiratory Time

In swimmers FIVC, FIV1, and PIF increased after exercise (median change: 23, 45, and 17 pp; *p* < 0.001 for each variable), and in water polo players they did not change significantly (*p* = 0.351, *p* = 0.596, and *p* = 1.000). The group × time interaction was significant for all three indices (*p* < 0.001 for each of them) (Table 3). The resting values of these indices were lower in swimmers than in water polo players (medians: FIVC 60% and 102%, FIV1 46% and 85%, PIF 32% and 52%). After exercise FIV1 and PIF did not differ between the groups, and FIVC remained lower in swimmers (*p* = 0.016).

FET shortened in both groups (swimmers *p* = 0.011; water polo players *p* < 0.001), and the shortening was greater in water polo players (median change: water polo players −31 pp, swimmers −5 pp; interaction p = 0.007). FET was longer in water polo players than in swimmers both at rest and after exercise (*p* < 0.001 at both time points). FEV_1_/FEV6 increased in both groups (swimmers *p* = 0.004; water polo players *p* = 0.036), without a significant interaction (*p* = 0.533), and at both time points, it was higher in swimmers. FEV3/FVC did not change in swimmers (*p* = 0.465), and in water polo players it increased (*p* < 0.001; interaction *p* = 0.001), remaining lower after exercise than in swimmers (*p* < 0.001).

### 3.3. Agreement of the Changes in MVV and FEV_1_ in Individual Athletes (Exploratory Analysis)

**Figure 1:**
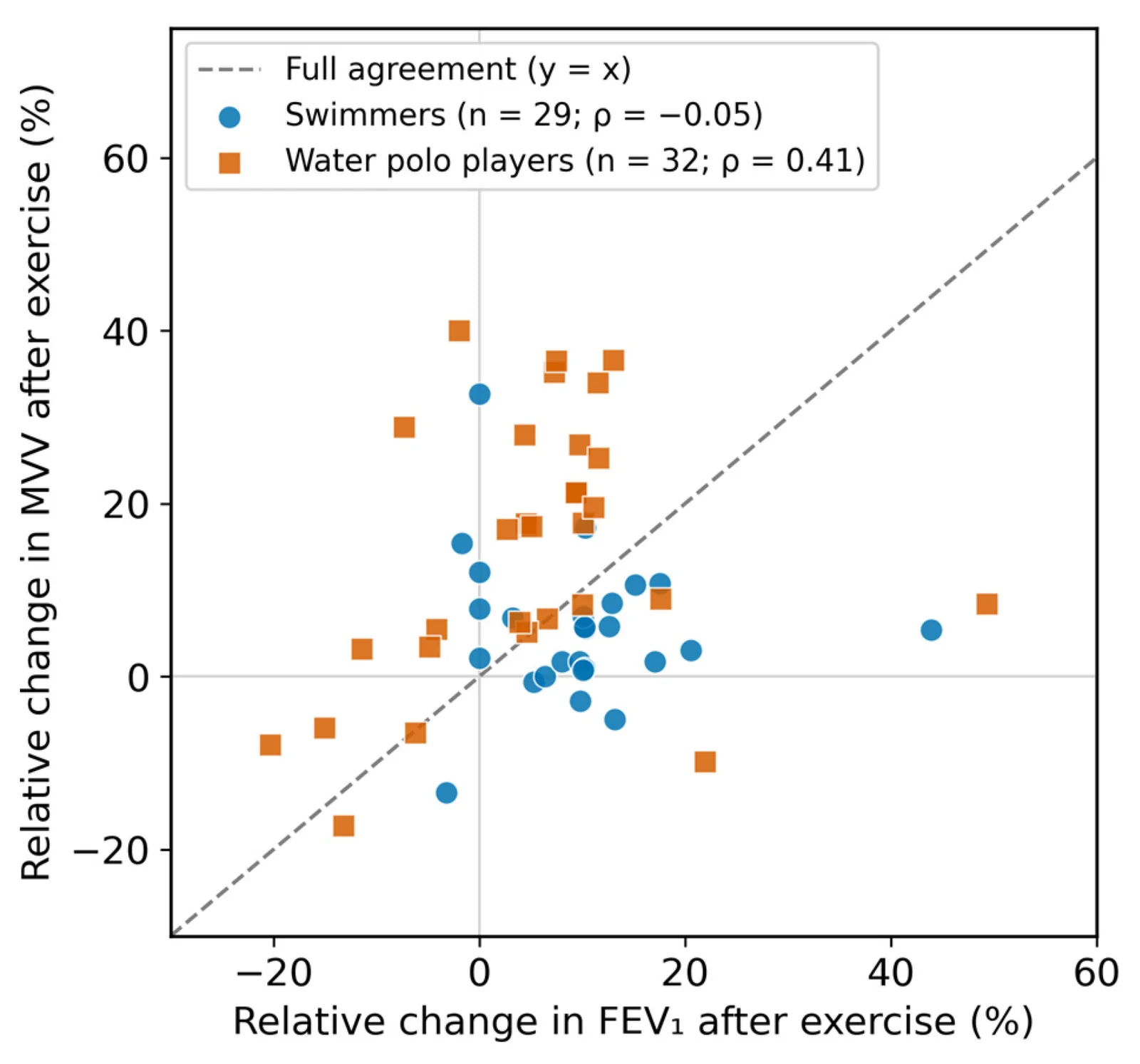
Relative change in MVV versus relative change in FEV_1_ after exercise in individual athletes. Dashed line: full agreement. ρ, Spearman’s correlation coefficient (for relative changes).

The change in MVV after exercise was not associated with the change in FEV_1_ in swimmers (Spearman’s ρ = 0.00; *p* = 0.98; *n* = 29), whereas in water polo players the association was moderate (ρ = 0.44; *p* = 0.012; *n* = 32) (Figure 1). In 10 of 29 swimmers and 7 of 32 water polo players the changes in MVV and FEV_1_ had opposite directions or one of them was zero.

**Figure 2:**
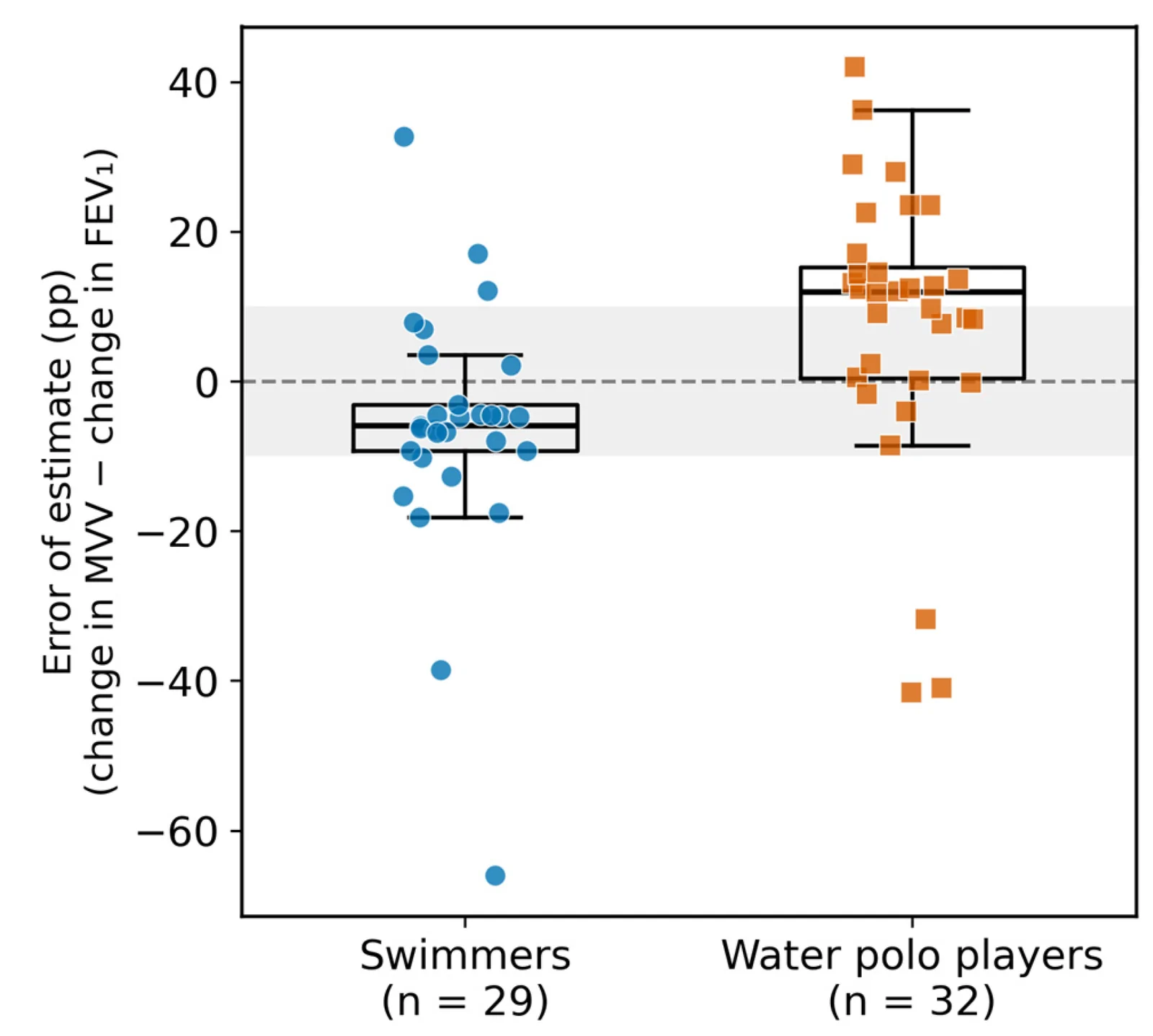
Error of the relative change in MVV estimated from FEV_1_ (change in MVV − change in FEV_1_, pp). Negative value: FEV_1_ overestimates the increase in MVV; positive: underestimates it. Grey background: ±10 pp.

The median error of the relative change in MVV estimated from FEV_1_ was −5.9 [−9.3 to −3.1] pp in swimmers (95% CI of the median −8.0 to −4.5), and 11.9 [0.5–15.2] pp in water polo players (95% CI 7.7 to 13.7); difference between the groups *p* < 0.001 (Figure 2). The error exceeded 10 pp (in both directions) in 10 of 29 swimmers and 20 of 32 water polo players, and the limits of agreement were from −40 to 27 pp in swimmers and from −29 to 45 pp in water polo players. The change in FEV_1_ did not predict the change in MVV after adjustment for group and resting MVV (*b* = 0.29; 95% CI −0.06 to 0.65; *p* = 0.107).

The sensitivity analysis in men only gave a similar picture: in swimmers (*n* = 18) ρ = −0.31 (*p* = 0.21), median error −5.3 [−9.3 to −4.4] pp, in water polo players ρ = 0.44, error 11.9 [0.5–15.2] pp; difference in the error between the groups *p* = 0.001, and the difference in the increase in MVV after adjustment for the resting value was not significant (*b* = 1.4 pp; 95% CI −8.8 to 11.7; *p* = 0.78). Among swimmers no sex difference was shown in the error of the estimate (*p* = 0.91), the increase in MVV (*p* = 0.28) or FEV_1_ (*p* = 0.33), with small group sizes (18 men, 11 women). In the model of the error with group, age, and BMI, the difference between the groups was 9.4 pp (95% CI −2.2 to 20.9; *p* = 0.11) and was not statistically significant.

### 3.4. Additional Exploratory Analyses

The estimate of the change in MVV from FEV_1_ was within ±10 pp of the actual change in 19 of 29 swimmers (66%; Wilson 95% CI 47–80%) and 12 of 32 water polo players (38%; 23–55%); Fisher’s test *p* = 0.041. The resting MVV/FEV_1_ ratio (both values in % of predicted) was lower in water polo players (0.95 [0.86–1.01]) than in swimmers (1.16 [1.05–1.21]; *p* < 0.001; in the analysis of men only *p* < 0.001), and the difference persisted after adjustment for age and BMI (*b* = −0.16; 95% CI −0.30 to −0.02; *p* = 0.025). The change in MVV in swimmers was associated with the change in FVC (ρ = 0.54; *p* = 0.003; after Benjamini–Hochberg correction in a family of 26 correlations *q* = 0.069), whereas in water polo players none of the changes in the remaining indices was associated with the change in MVV after correction (smallest *q* = 0.16). A relative fall in FEV_1_ of at least 10% in the early post-exercise period occurred in 0 of 29 swimmers and 4 of 32 water polo players, and an increase of at least 10% in 17 and 9 (Fisher *p* = 0.021).

## 4. Discussion

After 30 s of tethered swimming at maximal intensity, MVV increased both in swimmers and in water polo players. The increase was larger in water polo players whose resting MVV was lower, but after adjustment for the resting value, this difference was not significant. The increase in MVV was accompanied in the two groups by different changes in other indices. In swimmers, FEV_1_ and the inspiratory loop indices (FIVC, FIV1, PIF) increased, and in water polo players, FET shortened to a greater extent and the FEV3/FVC ratio increased. These results are consistent with the adopted hypothesis.

### 4.1. Changes in Spirometric Indices in Swimmers

In swimming, inspiration must be performed quickly, in the short phase allotted for breathing and repeated in every movement cycle over the whole distance. This loads the inspiratory muscles, as evidenced by their fatigue found after swimming exercise [7,8]. Swimmers also increase exercise ventilation mainly by increasing tidal volume [11], and inspiratory muscle training increased MVV and FIV1 in them [23]. However, the inspiratory loop indices should be interpreted very cautiously. At rest the median FIVC/FVC in swimmers was 0.52, and in 93% of the participants it was lower than 0.8, whereas in healthy people, FIVC is close to FVC. After exercise this ratio was 0.70, and FIV1 and PIF no longer differed between the groups. This suggests incomplete inspiratory effort in the resting measurement (the cause was not established) rather than a real improvement in inspiratory muscle function. Because all participants had the maneuvers explained, were able to practice them, and had undergone spirometry before, a learning effect is a less likely explanation, although it cannot be completely excluded. The increase in FIVC, FIV1, and PIF therefore does not justify a conclusion about the performance of the inspiratory muscles, and this hypothesis remains unverified. The increase in FEV_1_, FEV_1_/FVC, and the FEF50 and FEF75 flows in swimmers is consistent with the post-exercise bronchodilation described in healthy people [4,6].

### 4.2. Changes in Spirometric Indices in Water Polo Players

In water polo the opportunities for inspiration are irregular and short, and breathing is interrupted by periods of breath holding. The large shortening of FET and the increase in FEV3/FVC indicate that after exercise the water polo players performed the forced expiration faster than at rest. However, resting FET was considerably longer in this group than in swimmers, so the greater shortening could have resulted partly from the baseline value. Similarly, the larger increase in MVV in water polo players occurred with a lower resting value, and after adjustment for the resting value, it did not differ significantly from the increase in swimmers. After exercise the difference between the groups decreased but did not disappear. Because MVV depends on the breathing frequency achieved and on the volume of a single breath, it can be supposed that in swimmers the increase in MVV was associated mainly with a larger inspiratory volume and in water polo players with a shorter expiratory time. This interpretation is a hypothesis and requires verification with the use of measurement of respiratory pressures and analysis of the breathing pattern during play and racing.

### 4.3. Measured MVV and MVV Calculated from FEV_1_

Calculating MVV from FEV_1_ assumes a constant relationship between bronchial patency and ventilatory capacity [1,3]. MVV calculated as the product of FEV_1_ and a constant factor therefore changes to the same degree as FEV_1_. In water polo players the median relative change in FEV_1_ was 4.8% (non-significant change, *p* = 0.121), and that in measured MVV was 17.2%; so calculated MVV would underestimate the post-exercise increase in ventilatory capacity. In swimmers the relationship was the reverse: FEV_1_ increased by 10.2% and measured MVV by 5.6%, and so the calculated MVV would overestimate this increase. Therefore, in neither group did the change in FEV_1_ reflect the change in MVV, and the direction of the error differed between the groups. This is confirmed by the change in the MVV/FEV_1_ ratio, which after exercise decreased in swimmers and increased in water polo players. This indicates that in the early post-exercise period (within approximately 2 min) the assumption of a constant relationship between FEV_1_ and MVV is not met. This conclusion concerns only the early post-exercise phase. In the following minutes, bronchodilation gradually subsides, and in persons with bronchial hyperresponsiveness, frequent in swimmers, bronchoconstriction with a fall in FEV_1_ may occur between the 5th and the 15th minute after exercise [13,19]. The relationship between FEV_1_ and MVV may therefore change during recovery, and its course requires assessment at subsequent time points. Moderate agreement of measured and calculated MVV was described previously at rest [2]. Already at rest the MVV/FEV_1_ ratio varies between individuals [24], and the equation developed for athletes explained about 60% of the variability of MVV [3]. These equations were derived from resting measurements, so they should not be used immediately after exercise. The factors that may increase MVV independently of bronchial patency include recruitment and coordination of the respiratory muscles and greater voluntary effort during a test performed shortly after exercise. The work of the respiratory muscles during maximal exercise accounts for a considerable part of oxygen uptake, and its increase affects the distribution of blood flow between the respiratory muscles and the lower limbs [9,10]. On the basis of our data, it cannot be determined which of these mechanisms was of importance.

The results of the individual analysis strengthen this conclusion. In swimmers, the change in FEV_1_ carried no information about the change in MVV (ρ = 0.00), and in water polo players, the association was only moderate (ρ = 0.44), and so even for a correctly determined direction of the error, there is no coefficient that would make it possible to correct the calculated value. The limits of agreement were wide in both groups (see above). The difference in the direction of the error between the disciplines remained visible in men only, but after adjustment for age and BMI it was not significant (95% CI −2.2 to 20.9 pp), which does not allow it to be attributed to the discipline alone: the groups differ in age, BMI, and training volume, and the confidence interval is wide. Lack of significance is not evidence of lack of a difference.

The lower resting MVV/FEV_1_ ratio in water polo players, persisting after adjustment for age and BMI and in the analysis of men only, indicates that ventilatory capacity measured in a voluntary test is not, in them, a simple function of bronchial patency already at rest. Possible explanations (breathing pattern, coordination of the respiratory muscles, experience in breathing maneuvers) were not verified, and the ratio calculated from values in % of predicted is not directly comparable with the classical MVV/FEV_1_ (35–40). The association of the change in MVV with the change in FVC, and not FEV_1_, in swimmers is consistent with the hypothesis that their increase in MVV depends mainly on tidal volume, but it did not survive with certainty the correction for multiple testing (*q* = 0.07) and requires confirmation. The relative fall in FEV_1_ of at least 10% in some of the water polo players in the early post-exercise period may reflect early bronchoconstriction or differences in the technique of the maneuver; on the basis of a single measurement this cannot be determined.

FVC, PEF, and FEF25 did not change significantly in either group. The values of these indices change between consecutive trials even without intervention, which is attributed to the effect of learning the maneuver [25,26]. The lack of changes may also result from the fact that short exercise in tethered swimming affects these indices less than other types of exercise.

Swimming training is associated with a load on the airways. Bronchial hyperresponsiveness is found more often in swimmers than in athletes of other disciplines [13], and chronic exposure to the by-products of water disinfection in swimming pools favors inflammatory changes of the airway mucosa [27,28]. Changes in lung function in the course of swimming training have been described both in healthy athletes and in athletes with allergic diseases [29]. These are chronic phenomena, separate from the acute response to exercise analyzed in the present study. In the same group of swimmers, after identical exercise, an increase in peak nasal inspiratory flow was found which was not associated with the severity of changes in the paranasal sinuses in cone-beam computed tomography (CBCT) [30]. This indicates that post-exercise changes in the patency of the upper and lower airways may proceed independently of each other in the same persons.

### 4.4. Practical Significance

The results argue against replacing measured MVV with the value calculated from FEV_1_ in the assessment of the ventilatory capacity of water sports athletes in the early post-exercise period (within approximately 2 min). In water polo players the calculated value would underestimate and in swimmers overestimate the post-exercise increase in MVV. If ventilatory reserve is to be assessed in this period, direct measurement in the 12 s maximal voluntary ventilation test appears preferable to the value estimated from FEV_1_, which some spirometers give automatically. Whether this has clinical implications requires further study. The results do not allow inference about later periods of recovery.

### 4.5. Limitations

The study was cross-sectional in character. The groups differed in age and sex composition (the water polo group consisted only of men, and among the swimmers, there were 11 women), as well as in body mass and BMI, while the difference in height was not statistically significant (Table 1). The predicted values took into account sex, age, and body height, and the magnitude of post-exercise bronchodilation does not differ between women and men [6]; however, an effect of sex on other elements of the response to exercise cannot be excluded, and the differences between the groups may partly reflect differences in their composition rather than the discipline itself. Both groups were examined in the same phase of the training cycle (competitive period, i.e., at the stage of peak competitive preparation) with the use of an identical exercise protocol, which limits the influence of differences in training status between the groups; however, differences in training volume and type between the disciplines remain. The post-exercise measurement was performed at only one time point (the FVC test started approximately 20–30 s after exercise and the MVV test immediately after it; the whole measurement was completed within approximately 2 min), so it is not known whether the observed changes persist in further recovery or how they change in the period in which exercise-induced bronchoconstriction may develop (5–15 min). The time of the individual maneuvers was not recorded separately, so the timing of the post-exercise measurement is approximate, and the FVC and MVV values were obtained at slightly different moments of early recovery. In further studies it would be advisable to perform measurements of FEV_1_ in the 1st, 5th, 10th, 15th, and 30th minute and of MVV in the 1st and 10th minute after exercise. This would make it possible to assess the course of the relationship between FEV_1_ and MVV during recovery and to detect exercise-induced bronchoconstriction, while limiting the number of MVV tests would reduce the risk of respiratory muscle fatigue and of the influence of hyperventilation itself on bronchial patency. Respiratory muscle strength was not measured. The MVV test was performed only once at each time point, so its within-session repeatability could not be assessed. The spirometric measurement in the competitive period was performed in one measurement session, which was not repeated on another day, and so it is not possible to assess between-day within-subject variability. Although all participants were familiarized with the maneuvers before the measurements, a learning effect cannot be completely excluded. At rest FIVC in swimmers was considerably lower than FVC (see above), and FEV3/FVC reached in them the upper ceiling of 105%; thus the inspiratory indices and FEV3/FVC have limited interpretative value. Spirometric screening against the lower limit of normal was not used as an exclusion criterion, and so undiagnosed airway obstruction or bronchial hyperresponsiveness in individual participants cannot be excluded. The data on the inspiratory indices are incomplete in water polo players (*n* = 15–16). The analysis of agreement of MVV and FEV_1_ was exploratory, not planned before data collection and without correction for multiple testing, and the MVV and FEV_1_ values were expressed as percentages of predicted values, so the error concerns relative changes, not absolute ventilation. The results concern competitive swimmers and water polo players examined in the early post-exercise period (within approximately 2 min) after 30 s of maximal tethered swimming, and they should not be extrapolated to female water polo players, other populations, other exercise protocols, or later periods of recovery.

## 5. Conclusions

1. After 30 s of tethered swimming at maximal intensity, MVV increases both in swimmers and in water polo players, and the larger increase in water polo players is associated with the lower resting value.
2. In this exploratory study, under this exercise protocol, the increase in MVV in swimmers was accompanied by an increase in FEV_1_ and in water polo players by a shortening of forced expiratory time without a significant change in FEV_1_; the increase in the inspiratory loop indices in swimmers results to a large extent from incomplete inspiratory effort in the resting measurement and does not prove an improvement in inspiratory muscle function.
3. In the early post-exercise period (within approximately 2 min), the change in FEV_1_ does not reflect the change in MVV in individual athletes: MVV estimated from FEV_1_ would overestimate the increase in swimmers and underestimate it in water polo players, with wide limits of agreement. In this period, direct measurement of MVV in the 12 s test appears preferable to the estimated value. The clinical implications of this finding, the course of this relationship in further recovery, and the role of sex, age, and BMI require further research.

## Figures and Tables

**Table 1:** Characteristics of the participants and comparison of swimmers and water polo players (mean ± SD or n (%)).

Variable	Swimmers, All (*n* = 29)	Swimmers, Men (*n* = 18)	Swimmers, Women (*n* = 11)	Water Polo Players, Men (*n* = 33)	*p*, Swimmers vs. Water Polo Players
Sex, men, *n* (%)	18 (62.1)	18 (100.0)	0 (0.0)	33 (100.0)	<0.001
Sex, women, *n* (%)	11 (37.9)	0 (0.0)	11 (100.0)	0 (0.0)	
Age (years)	21.0 ± 3.2	21.3 ± 3.7	20.4 ± 2.2	23.4 ± 5.0	0.027
Body height (cm)	180.9 ± 8.2	185.6 ± 6.2	173.2 ± 4.2	184.5 ± 6.6	0.057
Body mass (kg)	73.3 ± 10.3	79.8 ± 6.1	62.8 ± 6.1	82.6 ± 9.8	0.003
BMI (kg/m^2^)	22.3 ± 2.0	23.2 ± 1.3	21.0 ± 2.3	24.3 ± 2.5	0.002

Water polo players: *n* = 33 in the table (anthropometric data), *n* = 32 in the spirometric analysis. Sex: Fisher’s exact test; continuous variables: Mann–Whitney U test (mean ± SD are presented for descriptive purposes). BMI, body mass index; SD, standard deviation.

**Table 2:** Indices of the expiratory flow–volume curve and MVV at rest and after exercise in swimmers and water polo players (% of predicted value).

Variable	Swimmers, Rest (*n* = 29)	Swimmers, Post-Exercise	Water Polo Players, Rest	Water Polo Players, Post-Exercise	Group *p*	Time *p*	Group × Time *p*
FVC	117 (106–127)	116 (105–129)	120 (113–136)	121 (112–131)	0.101	0.752	0.598
FEV_1_	114 (103–119)	119 (114–133) *	113 (98–120)	114 (106–124)	0.215	<0.001	**0.033**
FEV_1_/FVC (*n* WP = 28)	99 (95–107)	106 (96–111) *	93 (86–99) †	92 (87–99) †	<0.001	0.042	0.132
PEF (*n* WP = 30)	93 (81–102)	98 (78–106)	90 (78–107)	91 (81–103)	0.594	0.633	0.449
FEF25 (*n* S = 28)	98 (88–104)	101 (82–109)	90 (80–95)	88 (82–96)	0.016	0.749	0.898
FEF50 (*n* WP = 31)	96 (89–106)	109 (97–128) *	95 (83–104)	91 (81–110) †	0.027	0.002	0.152
FEF75 (*n* WP = 27)	103 (92–122)	126 (103–158) *	89 (72–106) †	94 (78–112) *†	<0.001	<0.001	0.288
MVV	123 (116–130)	132 (118–141) *	106 (94–114) †	120 (102–136) *†	<0.001	<0.001	**0.048**

Data are presented as median (Q1–Q3) in percent of predicted value. The *p* values for the effects of group, time, and interaction: nonparametric analysis of variance for longitudinal data (ATS statistic, F1-LD-F1 design, df = 1). * *p* < 0.05 compared with rest in the same group (Wilcoxon test with Holm correction); † *p* < 0.05 compared with swimmers at the same time point (Mann–Whitney *U* test with Holm correction). Bold marks a significant group × time interaction. *n* S, *n* WP: number of persons with complete measurements, if smaller than in the group (FEF25: one value in a swimmer, considered a measurement artifact, was excluded).

**Table 3:** Indices of the inspiratory flow–volume loop and expiratory time at rest and after exercise in swimmers and water polo players (% of predicted value).

Variable	Swimmers, Rest (*n* = 29)	Swimmers, Post-Exercise	Water Polo Players, Rest	Water Polo Players, Post-Exercise	Group *p*	Time *p*	Group × Time *p*
FIVC (*n* WP = 16)	60 (48–81)	87 (74–99) *	102 (95–111) †	103 (96–107) †	<0.001	<0.001	**<0.001**
FIV1 (*n* WP = 15)	46 (37–57)	89 (74–117) *	85 (66–124) †	114 (76–122)	0.002	<0.001	**<0.001**
PIF (*n* WP = 18)	32 (24–38)	50 (37–71) *	52 (35–65) †	59 (39–65)	0.021	<0.001	**<0.001**
FET	37 (36–47)	33 (25–38) *	86 (78–92) †	57 (37–78) *†	<0.001	<0.001	**0.007**
FEV_1_/FEV6 (*n* WP = 30)	97 (92–105)	103 (93–110) *	89 (80–94) †	90 (85–99) *†	<0.001	<0.001	0.533
FEV3/FVC (*n* WP = 31)	105 (105–105)	105 (105–105)	102 (101–104) †	104 (103–105) *†	<0.001	<0.001	**0.001**

Data are presented as median (Q1–Q3) in percent of predicted value. The *p* values for the effects of group, time, and interaction: nonparametric analysis of variance for longitudinal data (ATS statistic, F1-LD-F1 design, df = 1). * *p* < 0.05 compared with rest in the same group (Wilcoxon test with Holm correction); † *p* < 0.05 compared with swimmers at the same time point (Mann–Whitney *U* test with Holm correction). Bold marks a significant group × time interaction. *n* WP: number of water polo players with complete measurements, if smaller than in the group. FEV3/FVC in swimmers at rest reached the value of 105% (upper ceiling) in 90% of the participants, so its interpretation is limited.

## Data Availability

The data presented in this study are available on request from the corresponding author due to institutional data protection requirements and participant confidentiality.
